# MicroRNA-99a inhibits tumor aggressive phenotypes through regulating HOXA1 in breast cancer cells

**DOI:** 10.18632/oncotarget.5355

**Published:** 2015-09-19

**Authors:** Xiaolong Wang, Yaming Li, Wenwen Qi, Ning Zhang, Mingjuan Sun, Qiang Huo, Chang Cai, Shangge Lv, Qifeng Yang

**Affiliations:** ^1^ Department of Breast Surgery, Qilu Hospital, Shandong University, Jinan, Shandong, 250012, P.R. China; ^2^ School of Medicine, Shandong University, Jinan, Shandong, 250012, P.R. China; ^3^ Department of Pathology Tissue Bank, Qilu Hospital, Shandong University, Jinan, Shandong, 250012, P.R. China

**Keywords:** MiR-99a, HOXA1, breast cancer, proliferation, invasion

## Abstract

MicroRNAs (miRNAs) are key regulators of tumor progression. Based on microarray data, we identified miR-99a as a potential tumor suppressor in breast cancer. Expression of miR-99a is frequently down-regulated in breast cancer tissues relative to normal breast tissues. Reduced miR-99a expression was highly associated with lymph node metastasis and shorter overall survival of patients with breast cancer. Gain- and loss-of-function studies revealed that, miR-99a significantly inhibits breast cancer cell proliferation, migration, and invasion. An integrated bioinformatics analysis identified *HOXA1* mRNA as the direct functional target of miR-99a, and this regulation was confirmed by luciferase reporter assay. Furthermore, we showed for the first time that HOXA1 expression is elevated in breast cancer tissues. Knockdown of HOXA1 significantly inhibited breast cancer cell proliferation, migration and invasion, and restoration of HOXA1 partially rescued the inhibitory effect of miR-99a in breast cancer cells. Collectively, our data indicate that miR-99a plays a tumor-suppressor role in the development of breast cancer, and could serve as a potential therapeutic target for breast cancer treatment.

## INTRODUCTION

Breast cancer is the most commonly diagnosed cancer and the leading cause of cancer death in women worldwide, accounting for 23% of total new cancer cases and 14% of total cancer death [1]. Although mortality from breast cancer has been remarkably reduced, metastasis remains the major obstacle to the treatment of this disease [2]. Breast cancer metastasis is a complex process, involving cell proliferation, local invasion, intravasation, survival in the systemic circulation, extravasation and finally successful colonization and outgrowth at secondary sites [3, 4]. To improve the prognosis of patients with breast cancer, novel effective screening strategies and treatments are needed.

MicroRNAs (miRNAs) are a class of endogenous, non-protein-coding RNA about 22 nucleotides in length. These small RNAs post-transcriptionally regulate the translation and degradation of mRNAs by base-pairing to the 3′ untranslated regions (UTRs) of target mRNAs [5]. Although the biological functions of miRNAs are not fully understood, emerging data indicate that aberrantly expressed miRNAs can regulate tumorigenesis by functioning as tumor suppressors or oncogenes [6, 7]. A subset of cancer-specific miRNAs has been identified in multiple types of cancers, including prostate cancer [8], lung cancer [9], gastric cancer [10], and brain cancer [11]. In previous studies, we demonstrated that in primary breast cancer tissues, expression of miR-30a was significantly lower than in paired normal tissues, and overexpression of miR-30a suppressed breast tumor growth and metastasis by targeting metadherin [12]. Furthermore, we demonstrated that miR-339–5p can strongly suppress the expression of MDM2 and increase the levels and function of p53 protein; consistent with these observations, forced expression of miR-339–5p inhibits the progression of colorectal cancers *in vitro* and *in vivo* [13]. Multiple studies have shown that miRNAs such as miR-21, miR-31, and miR-210 play critical roles in breast cancer initiation and progression [14–16]. However, the functional significance of miRNA dysregulation in breast cancer remains unclear.

In this study, we found that expression of miR-99a was significantly reduced in breast cancer tissues relative to normal breast tissues, and miR-99a down-regulation was associated with breast cancer progression. Inversely, overexpression of miR-99a inhibited breast cancer cell proliferation, migration, and invasion. Furthermore, we identified *HOXA1*, a putative oncogene in breast cancer, as a direct and functional target of miR-99a. Therefore, our study demonstrated that miR-99a suppresses the progression of breast cancer by inhibiting HOXA1 function.

## RESULTS

### MiR-99a expression is suppressed in breast cancer and is correlated with clinical features and prognosis of breast cancer patients

**Figure 1:**
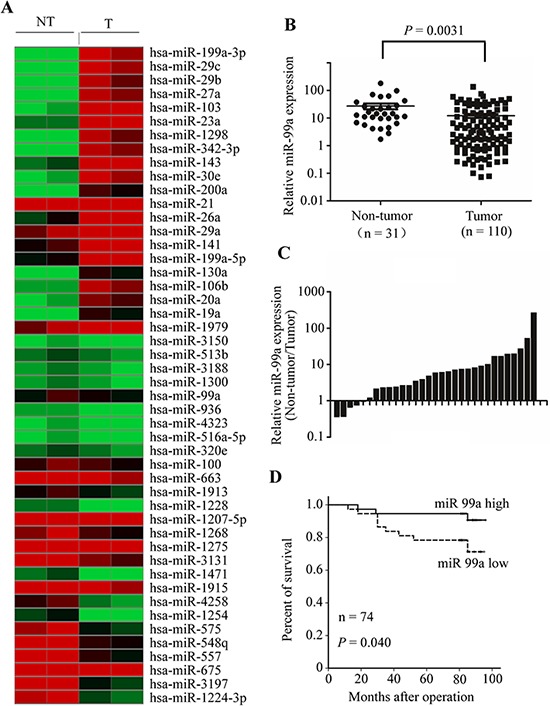
miR-99a expression is reduced in breast tumors and predicts better overall survival in breast cancer patients **A.** Heat map diagram depicting expression of 48 miRNAs dysregulated in breast cancer tissues compared with normal breast tissues. **B.** miR-99a expression levels, determined by qPCR. Each point represents a sample, and the bars represent means ± S. D. **C.** Comparison of miR-99a expression level between primary breast cancers and their corresponding non-tumorous tissues. **D.** Kaplan-Meier curves of patients with breast cancers. Patients were categorized into low- or high-expression groups. Low expression of miR-99a in breast cancer is associated with poorer prognosis.

To explore the role of miRNA in tumorigenesis of breast cancer, we first examined the miRNA expression profiles in breast cancer tissues. miRNA microarray was performed in two cases of tumor samples compared to matched benign breast tissues. We identified 21 miRNAs with significantly higher expression in breast cancer and 27 miRNAs with lower expression (Figure 1A). One of the down-regulated miRNAs was miR-99a, whose role in breast cancer was largely unknown; we focused on this miRNA in our subsequent investigations.

Next, we verified miR-99a expressions in breast tissue specimens using qPCR. Consistent with the array analysis, miR-99a expression was significantly down-regulated in tissues from 110 cases of primary breast cancer, relative to 31 samples of normal breast tissue (*P* = 0.0031, Figure 1B). Additionally, as shown in Figure 1C, in 84% (26 of 31) of breast cancers, miR-99a expression was reduced relative to the corresponding non-tumorous breast tissues from the same patients. Moreover, the expression levels of miR-99a were also reduced in five breast cancer cell lines, relative to those in the immortalized normal mammary epithelial cell line, MCF10A (Supplementary Figure S1).

To determine the prognostic significance of miR-99a down-regulation in breast cancer, we analyzed the correlation between miR-99a expression and patient survival. Low miR-99a expression was significantly associated with shorter overall survival (*P* = 0.040, Figure 1D).

In addition, we analyzed the relationship between the expression of miR-99a and the clinicopathologic factors of breast cancer patients. MiR-99a expression was remarkably lower in breast cancer patients with tumor metastasis (*n* = 48) than in those without metastasis (*n* = 35) (*P* = 0.0353, Table 1). These results suggested that down-regulation of miR-99a may play an important role in the progression of breast cancer.

**Table 1:** Association of miR-99a expression with clinicopathologic factors of breast cancer patients

Clinicopathological variables	Number of cases	Median expression of miR-99a	*P*
Age			0.2360
≤48	42	167.3 ± 50.02	
>48	41	101.1 ± 22.86	
Histology			0.7016
Ductal	73	109.8 ± 23.75	
Lobular	4	52.28 ± 33.97	
Other	6	160.4 ± 87.40	
Tumor size (cm)			0.2519
≤2	26	147.8 ± 53.74	
>2	57	93.71 ± 20.22	
Nodal status			0.0353
Negative	35	164.2 ± 44.59	
Positive	48	71.65 ± 17.57	
ER			0.4625
Negative	11	73.12 ± 41.82	
Positive	62	109.1 ± 19.09	
PR			0.9866
Negative	14	103.0 ± 39.55	
Positive	59	103.8 ± 19.47	
HER-2			0.3051
Negative	70	107.4 ± 17.96	
Positive	3	17.11 ± 4.348	

### miR-99a inhibits breast cancer proliferation, migration and invasion

**Figure 2:**
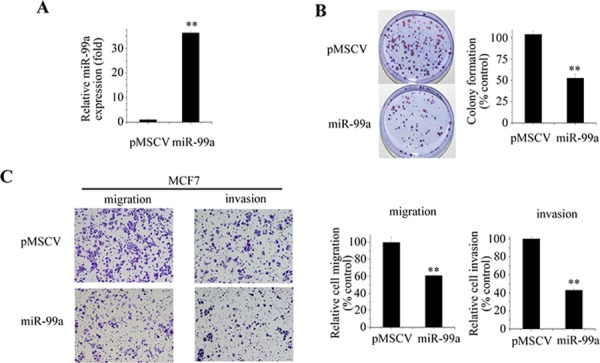
miR-99a inhibits aggressive behaviors of breast cancer cells **A.** Successful overexpression of mature miR-99a was confirmed by qPCR. Values of miR-99a expression were calculated as fold change relative to vector control. **B.** Effect of miR-99a on the clonogenic capacity of MCF7 cells. **C.** Transwell migration and invasion assays revealed that MCF7 cells stably transfected with miR-99a had lower migratory and invasive potentials. Cells were counted after staining with crystal violet. Representative images are shown on the left. Graphs indicated the average numbers of colonies and cells after quantitation and normalization against the control. Data represent means ± S.D. of at least three independent experiments. *P < 0.05, **P < 0.01.

To better understand the biological functions of miR-99a, we stably transfected MCF7 cells with vectors expressing pre-miR-99a. The highly up-regulated expression of miR-99a was confirmed by qPCR (Figure 2A). Colony formation assay revealed stable overexpression of miR-99a significantly decreased the proliferation rate of MCF7 (Figure 2B).

Given that the expression of miR-99a was highly associated with the metastatic properties of breast cancer, we wondered whether miR-99a might play an important role in migration and invasion. To test this idea, we employed a Transwell assay to detect the migration and invasion abilities of breast cancer cells following miR-99a overexpression. As shown in Figure 2C, transfection with miR-99a significantly decreased the migration and invasion capabilities of MCF7 cells (*P* < 0.01). Similar results were also obtained in MDA-MB-468 cells (Supplementary Figure S2)

### Reduction of miR-99a expression promotes breast cancer cell proliferation, migration, and invasion

**Figure 3:**
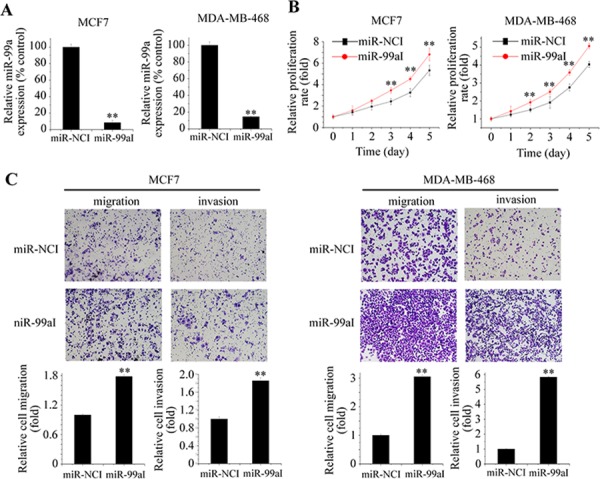
Inhibition of endogenous miR-99a promoted aggressive behaviors of breast cancer cells **A.** Suppression of miR-99a by specific inhibitor (miR-99aI) in MCF7 and MDA-MB-468 cells. MTT assay **B.** and Transwell assay **C.** revealed that miR-99a knockdown stimulated breast cancer cell proliferation, migration, and invasion. Data represent the means ± S.D. of at least three independent experiments. *P < 0.05, **P < 0.01.

To determine whether endogenous miR-99a regulates tumor progression, we transfected MCF7 and MDA-MB-468 cells with miR-99a inhibitor (miR-99aI) or miR inhibitor control (miR-NCI). Successful inhibition of endogenous miR-99a expression was confirmed by qPCR (Figure 3A). Inhibition of miR-99a significantly increased cell growth, migration, and invasion of breast cancer cells (Figure 3B and 3C), indicating that miR-99a suppresses breast cancer development by negatively regulating these processes.

### HOXA1 is a direct target of miR-99a

**Figure 4:**
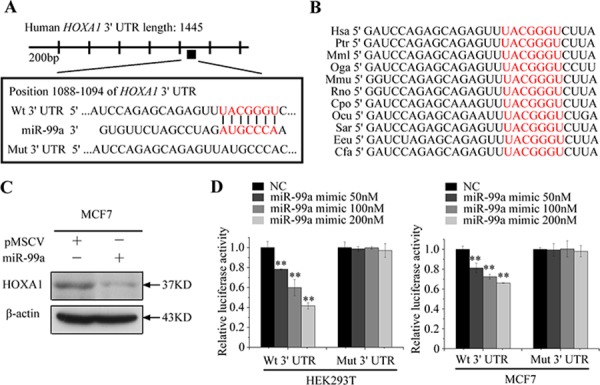
HOXA1 mRNA is a direct target of miR-99a **A.** Predicted targeting sequence of miR-99a at nucleotides 1088–1094 of the *HOXA1* 3′UTR. **B.** Sequence alignment of the predicted miR-99a seed region in the 3′UTR of *HOXA1* from 11 organisms. **C.** Western blot assay revealed that overexpression of miR-99a level significantly reduced HOXA1 expression in MCF7 cells. **D.** Relative luciferase activity was analyzed after reporter plasmids were cotransfected with miR-99a mimic or miR mimic control. Wt: wild type; Mut: mutant type. Data represent means ± S.D. of at least three independent experiments. *P < 0.05, **P < 0.01.

To explore the underlying molecular mechanism of miR-99a-mediated growth and metastasis suppression, we performed in silico studies to search for potential gene targets of miR-99a using the bioinformatics algorithms: TargetScan, PicTar, and miRanda (supplementary Table S2). All three algorithms predicted *HOXA1* as a target of miR-99a [20, 21]; the putative target sequence is in base pairs 1088–1094 of the *HOXA1* 3′UTR (Figure 4A). Homology searches revealed that this putative binding site is evolutionarily conserved (Figure 4B).

To further confirm that *HOXA1* is a direct target of miR-99a, we investigated whether overexpression of miR-99a resulted in reduced *HOXA1* expression. Western blot analysis confirmed that high expression of miR-99a significantly suppressed the level of endogenous HOXA1 protein (Figure 4C).

To determine whether miR-99a binds directly to the 3′UTR of target mRNA, we cloned the putative binding site of miR-99a into the pmiRGLO vector. For luciferase activity assays, miR-99a mimic was cotransfected with pmiRGLO-3′UTR vectors into HEK293T cells. As shown in Figure 4D, relative luciferase activity was remarkably decreased by miR-99a mimic when the wild-type 3′UTR of *HOXA1* was present. In addition, we constructed a mutant vector which contained the HOXA1 3′UTR with mutations in the putative miR-99a binding site (Figure 4A). MiR-99a mimic caused no obvious change in luciferase activity in cells transfected with the mutant 3′UTR vectors. Similar results were obstained in MCF7 cells (Figure 4D). Taken together, these findings indicated that *HOXA1* mRNA is a direct, down-stream target of miR-99a in breast cancer cells.

### HOXA1 is up-regulated in breast cancer and promotes breast cancer cell proliferation, migration, and invasion

**Figure 5:**
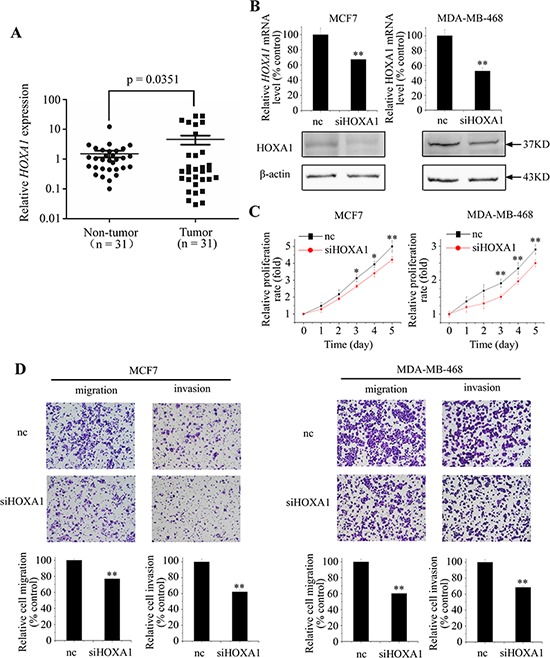
HOXA1 functions as an oncogene in breast cancer cells **A.** *HOXA1* expression levels in breast cancer tissues and normal breast tissues were analyzed by qPCR. **B.** Efficacy of RNA interference was verified by qPCR and western blotting. (C and D) Down-regulation of HOXA1 by si-HOXA1 inhibited cell proliferation, migration, and invasion in MCF7 and MDA-MB-468 cells. Data represent means ± S.D. of at least three independent experiments. *P < 0.05, **P < 0.01.

Previous reports indicated that HOXA1 plays an important role in cancer development [22, 23]. However, the biopathological significance of HOXA1 in human cancer, especially breast cancer, is still largely unknown. Therefore, we first tested the *HOXA1* mRNA levels in 31 breast cancer patient samples and their corresponding normal breast tissues. As shown in Figure 5A, *HOXA1* mRNA levels were significantly higher in breast cancer tissues than in adjacent normal tissues (*P* = 0.0351).

To better understand the functional role of HOXA1, we performed loss-of-function studies using small interfering RNA against HOXA1 (si-HOXA1). qPCR confirmed that transfection with si-HOXA1 could effectively reduce HOXA1 expression in both MCF7 and MDA-MB-468 cells (Figure 5B). Functional assays revealed that cell proliferation, migration, and invasion activities were significantly inhibited in si-HOXA1-transfected MCF7 and MDA-MB-468 cells, in comparison with si-control cells (Figure 5C and 5D). This phenotype was similar to that induced by overexpression of miR-99a.

### The tumor suppressor role of miR-99a is mediated by down-regulation of HOXA1

**Figure 6:**
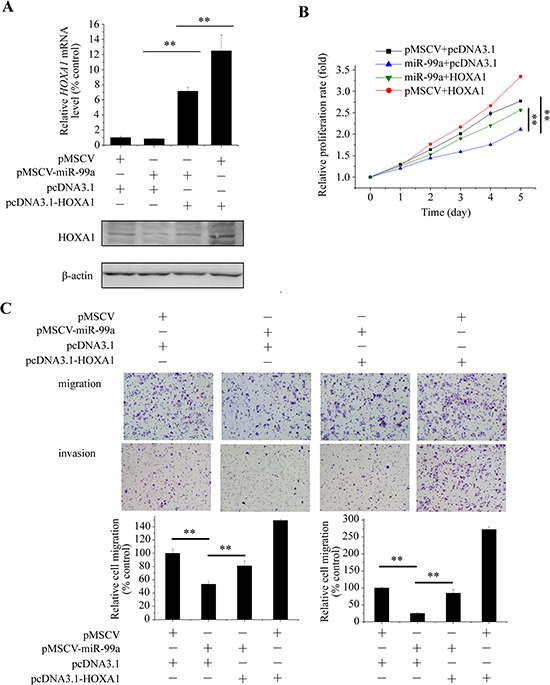
Tumor-suppressive effect of mir-99a is mediated by targeting of *HOXA1* mRNA MCF7 cells were transfected with pMSCV or miR-99a together with either HOXA1 or pcDNA3.1. **A.** mRNA and protein levels of HOXA1 were detected. MTT assay **B.** and Transwell assay **C.** were performed to examine proliferation, migration, and invasion abilities of the transfected cells, and relative migration and invasion rates were analyzed quantitatively. Data represent the means ± S.D. of at least three independent experiments. **P < 0.01.

To determine whether miR-99a-induced inhibition of cell proliferation, migration and invasion could be reversed by restoration of HOXA1 expression, we performed a gain-of-function assay. Specifically, we transfected a vector expressing *HOXA1* without its 3′UTR, which resulted in constitutive expression of HOXA1 without the potential for miR-99a-mediated degradation, into MCF7-miR-99a or MCF7-pMSCV cells. The mRNA and protein levels of HOXA1 was detected (Figure 6A). As shown in Figure 6B and 6C, overexpression of HOXA1 partially abrogated miR-99a-mediated suppression of proliferation, migration, and invasion in MCF7 cells. These results reinforced that *HOXA1* mRNA is a direct functional target of miR-99a.

## DISCUSSION

Over the past few years, multiple studies have strongly supported the involvement of miRNAs in the initiation and progression of tumors. Wu et al. found that miR-19a/b targets and down-regulates the tumor suppressor MXD1 in gastric cancer, and forced expression of miR-19a/b significantly increases migration *in vitro* and *in vivo*. Moreover, miR-19a/b is up-regulated in gastric cancer tissues, and its expression level is correlated with metastasis [24]. Wang et al. demonstrated that the expression level of miR-486–5p is lower in lung tumor samples, and is associated with tumor stage and lymph node metastasis of non-small-cell lung cancers. Reduced expression of miR-486–5p increases ARHGAP5 expression and enhances migration and invasion of cancer cells [25]. In addition, Zhou et al. declared that miR-105 secreted by cancer cells promotes tumor metastasis by targeting the mRNA encoding the tight junction protein ZO-1 [26].

MiR-99a, which belongs to the miR-99 family, is located in intron 13 of the C21orf34 gene at chromosome 21q [27]. Previous studies showed that miR-99a is frequently lost or expressed at a reduced level in various human cancers, including breast cancer [28, 29]. In addition, miR-99a is down-regulated in oral cancer. Up-regulation of miR-99a inhibits oral cancer migration and invasion [30], and overexpression of miR-99a in renal cell carcinoma induces cell-cycle arrest at G1 phase and suppresses tumorigenicity [31]. Higher levels of miR-99a inhibit the growth of prostate cancer cells and decrease the expression of prostate-specific antigen [32]. In addition, the expression level of miR-99a is correlated with overall survival rate in esophageal squamous cancer patients [33]. These results suggest that miR-99a is associated with tumor pathogenesis and development. However, the direct targets of miR-99a and its biologic functions in breast cancer remain poorly defined. In this study, we showed that miR-99a is down-regulated in breast cancer tissues, and that it inhibits the proliferation, migration and invasion activities of breast cancer cells. These data imply that miR-99a acts as a tumor suppressor in breast cancer.

Using three miRNA target prediction algorithms (TargetScan, PicTar and miRanda), we provided the first demonstration that *HOXA1* mRNA is a direct functional target of miR-99a in breast cancer. Because of the important role of miR-99a in many types of cancers, further studies will be needed to validate other miR-99a functional targets in cancers, which may facilitate development of possible clinical approaches for cancer treatment.

The HOX genes are members of a highly conserved subgroup of the homeobox transcription factor superfamily that play important roles in regulating cell fate, early developmental patterns and organogenesis [34–36]. Alterations in HOX genes are also associated with a variety of human cancers, including lung [37], breast [38], and hematological malignancies [39]. HOXA1, which was first identified in *Drosophila*, is a member of the HOXA group. In the human mammary gland, the expression of HOXA1 is very low or absent during normal growth and differentiation, but several studies have revealed its up-regulation in mammary carcinomas [40, 41]. Forced expression of HOXA1 in normal human mammary epithelial cells is sufficient to initiate oncogenic transformation and tumor formation *in vivo* [42]. In addition, HOXA1 promotes melanoma tumor growth and metastasis, and is associated with poor clinical outcome [22]. However, the biological function of HOXA1 in breast cancer remains poorly understood. Here, we demonstrate for the first time that HOXA1 was frequently up-regulated in breast cancer tissues. Furthermore, reduced expression of HOXA1 could inhibit breast cancer cell proliferation, migration and invasion. Restoration of HOXA1 expression rescued the inhibitory effect of miR-99a in breast cancer. These data suggested that the miR-99a/HOXA1 axis plays an important role in the regulation of breast cancer development.

In conclusion, we demonstrated that miR-99a could significantly inhibit breast cancer cell proliferation, migration and invasion. Conversely, overexpression of HOXA1, which is a direct functional target of miR-99a, promoted breast cancer cell growth and metastasis. The newly identified miR-99a/HOXA1 axis provides novel insight into the pathogenesis of breast cancer, and represents a potential therapeutic target for the treatment of breast cancer.

## MATERIALS AND METHODS

### Ethics statement and human tissue samples

All experimental procedures were approved by the Ethical Committee of Shandong University. Breast carcinoma and the adjacent normal tissues were obtained at the time of surgery from patients admitted to Qilu hospital from January 2004 to December 2011, and immediately stored at −80°C until use. All patients provided written informed consent for the use of these clinical materials in research.

### Cell lines and culture conditions

Human breast cancer cell lines MCF7, MDA-MB-468 and HEK293T were purchased from American Type Culture Collection (ATCC, Manassas, VA, USA), and were routinely maintained in DMEM/high glucose medium (Gibco-BRL, Rockville, IN, USA) supplemented with 10% fetal bovine serum (Haoyang Biological Manufacture, Tianjin, China), 100 U/ml penicillin and 100 μg/ml streptomycin in 5% CO2 at 37°C. The retroviral transient producer cell line H29 was cultured in complete DMEM medium as described above supplemented with tetracycline (1 mg/ml).

### Plasmid construction and transfection

The genomic region containing pre-miR-99a was amplified from human genomic DNA and cloned into vector pMSCV-puro. The resultant vector, pMSCV miR-99a or the control vector was transfected into H29 cells. After transfection, tetracycline was removed to allow production of retroviral vector. Two days later, retrovirus-containing medium was collected and stored at − 80°C. For *HOXA1* overexpression, vector was constructed as described previously [12]. Briefly, *HOXA1* cDNA was cloned into the multiple cloning sites of vector pcDNA3.1 (Invitrogen, Carlsbad, CA, USA), and the resultant expression vector and empty vector were transfected into MCF7 cells to establish HOXA1 overexpressing and control cell lines, respectively. Primers for plasmid construction are shown in the Supplementary Table. Transfection was performed with Lipofectamine 2000 (Invitrogen).

### Transfection with miRNA and small interfering RNA

MiR-99a mimic, miR mimic control, miR-99a inhibitor (miR-99aI), miR inhibitor control (miR-NCI), and small interfering RNA were purchased from GenePharma (Shanghai, China). Cells were seeded in 60 mm dishes (6 × 10^5^ cells/dish), transfected, and used for further assays.

### Quantitative real-time PCR (qPCR) analysis

Total RNA was extracted from cells using the TRIzol reagent (Invitrogen, Carlsbad, CA, USA). PrimeScript reverse transcriptase (RT) reagent kit (TaKaRa, Shiga, Japan) was used to synthesize cDNA from total RNA. MiRNA from 1 μg of total RNA was reverse transcribed using the Prime-Script miRNA cDNA Synthesis Kit (TaKaRa). Real-time PCR was performed on an Applied Biosystems StepOne Plus Real-Time PCR System. Primer information used in the study is provided in the Supplementary Table.

### Western blot analysis

Western blot analysis was performed as described previously [17]. In brief, cells were harvested and lysed in lysis buffer (1 × PBS, 1% NP40, 0.1% sodium dodecyl sulfate, 5 mM EDTA, 0.5% sodium deoxycholate and 1 mM sodium orthovanadate) containing protease inhibitors. Subsequently, 50 μg of total cellular protein from each sample were separated by 10% SDS-PAGE and electrotransferred onto polyvinylidene fluoride (PVDF) membrane using a semi-dry blotting apparatus (Bio-Rad, Hercules, CA, USA). The membranes were blocked with 5% nonfat milk at room temperature for 1 h, and then incubated with primary antibodies (Immuno-Way, Newark, DE, USA) overnight at 4°C. After incubation with the appropriate secondary antibodies, the protein bands were detected using the Pro-lighting HRP agent. Expression of β-actin was used as a loading control.

### MTT (3-(4,5-Dimethyl-2-thiazolyl)-2,5-diphenyl-2H-tetrazolium bromide) assay

MTT was purchased from Sigma. MTT assay was performed to measure the proliferation of cancer cells. In brief, cancer cells were seeded in 96-well plates in culture medium and incubated in 5% CO2 at 37°C. After incubation for the indicated time, 20 μl of MTT (5 mg/ml in PBS) was added into each well and incubated for 4 h. The supernatants were carefully aspirated, and 100 μl of dimethyl sulfoxide (DMSO) was added to each well. Absorbance values at 490 nm were measured on a Microplate Reader (Bio-Rad).

### Clonogenic assay

Cancer cells were plated in triplicate at a density of 1,000 cells per 60 mm plate and incubated for 18 days. The media were refreshed every 5 days. Colonies that formed were stained with 2% crystal violet (Sigma, St Louis, MO, USA) and counted. The stained colonies were photographed using an Olympus Live View Digital SLR camera (Olympus, Tokyo, Japan).

### Migration and invasion assays

Migration and invasion assays were performed using the Transwell system (24-wells, 8-μm pore size with polycarbonate membrane; Corning Costar, Lowell, MA, USA) as described previously [18]. The invasion assay was performed in the same manner as the migration assay except that the membrane was coated with Matrigel (BD Bioscience, SanJose, CA, USA). Briefly, cancer cells were starved in serum-free medium for 24 h at 37°C. Then, 100 μl of solutions containing 1 × 10^5^ cells were added to the upper inserts with or without pre-coated Matrigel. Cells attached to the lower surface were fixed with methanol and stained with 0.2% Giemsa. The successfully migrated cells were counted in five random fields using an Olympus light microscope.

### Dua-luciferase reporter assay

The dual-luciferase miRNA Target Expression vector pmirGLO (Promega, Madison, WI, USA) was used to generate luciferase reporter constructs. The 3′UTR of *HOXA1*, containing putative miR-99a-binding sites was amplified and cloned into pmirGLO. A mutant reporter plasmid was constructed as described previously [19]. Cells were seeded in 96-well plates and co-transfected with wild-type or mutated *HOXA1* 3′UTR constructs, and miR-99a mimic or negative control. Luciferase activity was measured with the dual-luciferase reporter assay system (Promega). *Firefly* luciferase activity was normalized against *Renilla* luciferase activity.

### Statistical analysis

All data are presented as means ± S.D. from at least three independent experiments. The software SPSS V18.0 was used for statistical analysis. Statistical significance of differences between two groups was evaluated using Student's *t*-test, and one-way ANOVA was used to determine the significance of differences among multiple groups. Differences with *P* < 0.05 were considered statistically significant.

## SUPPLEMENTARY FIGURES AND TABLES


