# Supplementary material for: MicroRNA-99a inhibits tumor aggressive phenotypes through regulating HOXA1 in breast cancer cells
Source: Oncotarget. 2015 Sep 19;6(32):32737–47. doi: 10.18632/oncotarget.5355 (PMC4741726; doi:10.18632/oncotarget.5355)
Supplement: Supplementary file 1 [file oncotarget-06-32737-s001.pdf]

SUPPLEMENTARY FIGURES AND TABLES

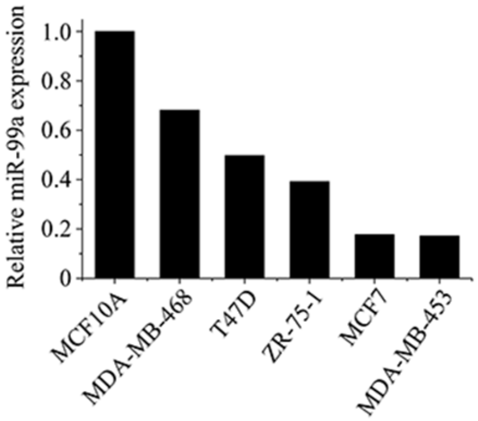

Supplementary Figure S1: Relative miR-99a expression levels in the immortalized normal mammary epithelial cell line, MCF10A, and breast cancer cell lines, MDA-MB-468, T47D, ZR-75-1, MCF7 and MDA-MB-453.

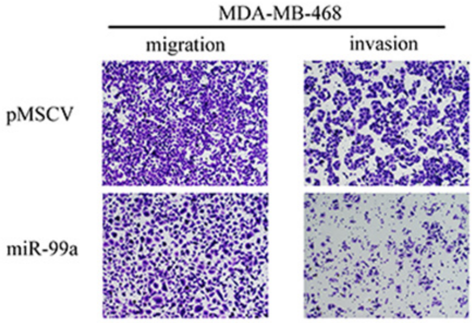

Supplementary Figure S2: Overexpression of miR-99a inhibited migration and invasion abilities in MDA-MB-468 cells. Representative images of three independent experiments were shown.

Supplementary Table S1: Primers for plasmid construction, and qPCR

	Name		Sequence
Plasmid construction			
	pMSCV miR-30a	F	ATACTCGAG TGAAACAAAAGCAGTTCGTGAAA
		R	GCAGAATTCTGTTGAGAATTGAAGCCTGCCTTGT
	pcDNA3.1-HOXA1	F	CCGGAATTCATGGACAATGCAAGAATGAACTC
		R	CCGCTCGAGTCAGTGGGAGGTAGTCAGAGT
	HOXA1 3'UTR Wt	F	CCGCTCGAGCGCACAATGTTTGATGTCCCCA
		R	TGCTCTAGAATTTACCACCCTTCTCCCTTTCC
	HOXA1 3'UTR Mut	F	TGGGCATAACTCTGCTCTGGATCATATCACTC
		R	ATGCCCACTTAGGATGTCTGTAATAAATAAATATACTC
qPCR			
	miR-99a	F	AACCCGTAGATCCGATCTTGTG
		R	GCGAGCACAGAATTAATACGAC
	HOXA1	F	TCCTGGAATACCCCATACTTAGC
		R	GCACGACTGGAAAGTTGTAATCC
	snRNA U6	F	GCTTCGGCAGCACATATACTAAAAT
		R	GCACGACTGGAAAGTTGTAATCC
	GAPDH	F	GGGCTGCTTTTAACTCTGGTAAAG
		R	CCATGGGTGGAATCATATTGG
	ACTIN	F	AGCGAGCATCCCCCAAAGTT
		R	GGGCACGAAGGCTCATCATT

Supplementary Table S2: Gene targets predicted by three bioinformatics strategies[a]

Name	GeneName	Position	TargetScan Sites	PicTar Sites	miRanda Sites
hsa-miR-99a-5p	NIPBL	chr5:37064420-37064426[+]	236[1]	236[1]	236[1]
hsa-miR-99a-5p	ZZEF1	chr17:3907885-3907891[-]	4[1]	4[1]	4[1]
hsa-miR-99a-5p	MBNL1	chr3:152181797-152181803[+]	1275[12]	1275[12]	2569[16]
hsa-miR-99a-5p	FZD5	chr2:208627869-208627875[-]	145[7]	145[7]	145[7]
hsa-miR-99a-5p	HS3ST3B1	chr17:14249260-14249267[+]	45[2]	45[2]	51[3]
hsa-miR-99a-5p	KBTBD8	chr3:67059188-67059195[+]	1721[18]	1721[18]	1721[18]
hsa-miR-99a-5p	TRIB1	chr8:126450121-126450127[+]	758[20]	758[20]	859[21]
hsa-miR-99a-5p	INSM1	chr20:20350863-20350869[+]	0[2]	0[2]	5[3]
hsa-miR-99a-5p	BAZ2A	chr12:56990825-56990832[-]	94[7]	94[7]	94[7]
hsa-miR-99a-5p	BAZ2A	chr12:56991781-56991787[-]	160[2]	160[2]	227[6]
hsa-miR-99a-5p	FGFR3	chr4:1809526-1809533[+]	7[3]	7[3]	14[3]
hsa-miR-99a-5p	EIF2C2	chr8:141541347-141541353[-]	331[8]	331[8]	339[9]
hsa-miR-99a-5p	SMARCA5	chr4:144474388-144474394[+]	1284[11]	1284[11]	1341[13]
hsa-miR-99a-5p	FZD8	chr10:35927723-35927729[-]	498[7]	498[7]	877[12]
hsa-miR-99a-5p	ICMT	chr1:6283496-6283502[-]	6[2]	6[2]	32[5]
hsa-miR-99a-5p	HOXA1	chr7:27132965-27132971[-]	17[1]	17[1]	34[1]
hsa-miR-99a-5p	AP1AR	chr4:113190442-113190449[+]	75[2]	75[2]	75[2]
hsa-miR-99a-5p	RAVER2	chr1:65296765-65296771[+]	65[4]	58[3]	65[4]
hsa-miR-99a-5p	MTMR3	chr22:30423581-30423587[+]	198[4]	198[4]	210[5]

(*Continued*)

Name	GeneName	Position	TargetScan Sites	PicTar Sites	miRanda Sites
hsa-miR-99a-5p	EPDR1	chr7:37991108-37991115[+]	56[2]	12[1]	956[5]
hsa-miR-99a-5p	SLC44A1	chr9:108151557-108151563[+]	1299[2]	1299[2]	1302[3]
hsa-miR-99a-5p	MTOR	chr1:11167241-11167247[-]	304[5]	304[5]	304[5]
hsa-miR-99a-5p	TRIB2	chr2:12882156-12882162[+]	145[9]	145[9]	145[9]

[a]The prediction was performed with starbase (http://starbase.sysu.edu.cn/).
